# The Influence of Printing Orientation on Surface Texture Parameters in Powder Bed Fusion Technology with 316L Steel

**DOI:** 10.3390/mi11070639

**Published:** 2020-06-29

**Authors:** Tomasz Kozior, Jerzy Bochnia

**Affiliations:** Department of Manufacturing Technology and Metrology, Kielce University of Technology, 25-314 Kielc, Poland; jbochnia@tu.kielce.pl

**Keywords:** additive manufacturing technology, powder bed fusion, 3D printing, surface geometrical parameters (SGP), lathe chuck jaws

## Abstract

Laser technologies for fast prototyping using metal powder-based materials allow for faster production of prototype constructions actually used in the tooling industry. This paper presents the results of measurements on the surface texture of flat samples and the surface texture of a prototype of a reduced-mass lathe chuck, made with the additive technology—powder bed fusion. The paper presents an analysis of the impact of samples’ orientation on the building platform on the surface geometrical texture parameters (two-dimensional roughness profile parameters (*Ra*, *Rz*, *Rv*, and so on) and spatial parameters (*Sa*, *Sz*, and so on). The research results showed that the printing orientation has a very large impact on the quality of the surface texture and that it is possible to set digital models on the building platform (parallel—0° to the building platform plane), allowing for manufacturing models with low roughness parameters. This investigation is especially important for the design and 3D printing of microelectromechanical systems (MEMS) models, where surface texture quality and printable resolution are still a large problem.

## 1. Introduction

### 1.1. 3D Printing

Additive manufacturing technologies were developed in the latter part of the 20th century, when the first additive technology was introduced—stereolithography [1]. In the subsequent years, we could notice the dynamic development of additive production technologies [2], manifested in many patent publications, such as FDM (fused deposition modeling) [3], SLS (selective laser sintering) [4], LOM (laminated object manufacturing) [5], SLM (selective laser melting) [6], and 3DP (3D printing) [7]. There are several ecological, environmental friendly additive manufacturing technologies such as FEF (freeze-form extrusion fabrication) [8,9].

Rapid prototyping of tools used in conventional production technologies such as machining, casting [10,11,12], or injection moulding allows for immediate subsequent printing necessary after testing. Moreover, additive technologies enhance optimization of production and enable fast adaptation of new solutions. Together with development of the technology, the increased precision of manufactured devices, as well as progress in laser technology, a dynamic evolution of additive technologies applications took place. In particular, this applies to powder-based technologies such as SLM, SLS, laser engineered net shaping (LENS), and so on. In many cases, studies confirm that the manufactured models can be characterized by significant density and ideal mechanical properties. Owing to the lack of uniform procedures concerning the studies that are available in the case of conventionally manufactured models, the development of additive technologies has partially decelerated. Owing to the layered nature of the manufacturing process, as presented above, with different methods of construction and connection of layers, the properties of the manufactured models depend on the correct selection of technological parameters [13] and further finishing processes [14]. There are numerous studies aimed at the determination of the impact of technological manufacturing processes parameters on properties and accuracy of manufactured models, as presented in the following publications [15,16,17,18,19,20]. Additive technologies can be classified based on many criteria [21], one of which is the type of input material used. In the case of this kind of classification, one differentiates the following technologies: powder-based (SLS, SLM, 3DP, and so on), liquid-based (SLA - stereolitography, PJ - PolyJet, PJM – PolyJet Matrix, MJM – Multi Jet Modeling, DODJET, FEF - freeze-form extrusion fabrication, and so on), and solid state material (FDM, FFF (fused filament fabrication), LOM, and so on) [22]. Each of the above-mentioned methods has its own advantages and drawbacks that will depend on the method of connecting layer and the delivery of the model and auxiliary material. Therefore, the application of the mentioned technologies differs as well. The following part of the paper explores additive technologies that use metal powder as a starting material. Within the last decade, a new type of additive production has been advanced, especially using FDM technology consisting of the construction of a physical object on other pre-existing models [23,24,25,26].

Additive technologies in the case of rapid tooling applications are broadly used for construction of casting moulds and models, 3D sand mold printing technology [27,28]; injection moulds, SLM and SLS (metal); and the printing of tools of complicated shapes (such as needed in the medical industry). Moreover, additive technologies using metal-based materials are commonly applied for the production of prototypes and fully functional elements in the automotive and aviation industries [29,30,31] as well as in robotics [32]. Current metal powder-based technologies are widely used in the odontology industry, where it is not necessary to construct large, rather but strong and corrosion-resistant dentures [33,34,35]. The medical industry pays even more attention to the application of additive technologies. This is highly related to the option to use three-dimensional models obtained directly from X-rays and computer tomography. Additive manufacturing technologies are also used in the food industry [36].

### 1.2. Literature Review

MEMS (microelectromechanical systems) is a potential application of 3D printing technology using metal-based materials [37,38,39]. In this application, models produced with 3D printing technologies, from both metal-based materials and plastics, must be characterized by appropriate surface quality and dimensional accuracy. The parameters of the surface layer obtained by additive technologies are largely influenced by technological parameters (e.g., grain size, layer thickness, printing direction, temperature, laser power, laser speed, cooling time) and the resolution of selected machines/3D printers. In the paper [37], the authors presented the possibilities of using 3D printing technology and selected machines for building MEMS models. The article mainly analyzes materials based on plastics and describes the disadvantages and advantages of currently used machines/printers in the context of the use of 3D printing to build MEMS models. It seems that increasing the accuracy of 3D printers is a natural consequence of their technological development, which will definitely increase the practical potential and uses 3D printing in MEMS production.

Studies related to the determination of geometrical texture quality of a model manufactured using both conventional and generative technologies utilizing strong, corrosion-resistant materials have been described in a few research papers.

In the papers of [40,41,42], different measurement systems have been used in order to analyse the measurement technology impact on the obtained results. The authors used both contact and optical measurement methods, considering X-ray computer tomography and multiscale 3D curvature analysis.

The authors of [43] carried out studies on models made using SLM technology and the powder AlSi10Mg. In the designed model, they determined basic parameters of geometrical texture on various surfaces oriented under variable angles in relation to the building platform. A measurement of the selected model dimensions was also performed.

Studies consisting of the analysis of geometrical texture quality of the models made using metal powders and SLM technology as well as the analysis of measurements methodology have been described in a few research works by [44,45,46].

The presented studies, based on the analysis of the impact of model orientation on the building platform on their SGPs (surface geometrical parameters), present actual application of the additive technologies in the construction of tools used in conventional methods of production, which must satisfy specified quality requirements of the top layer. The technological top layer plays a key role in the wearing process of mating machine parts. Additionally, it is responsible for a series of functional properties that condition the correct operation of the designed mechanisms. This is very important in the case of manufacturing prototypes, which are subjected to numerous tests that require the implementation of further design changes. Moreover, the quality of the top layer determines the further processes of model cleaning and type of finishing. It is also important that, in many cases, such as the production of elements of complicated internal shapes (e.g., turbine blades with complicated cooling channels), there is no option to perform finishing, which proves the presented studies concerning the impact of model orientation during construction to be considered as reasonable.

The influence of finishing on the surface roughness is presented in [47]. The authors of this work come to the fundamental conclusion that the finishing treatment significantly improves the surface roughness in relation to the surface obtained through additive technology and recommends finishing treatment after the additive shaping process. The results of roughness measurements for samples made with SLM technology and then machined mechanically (finish machining), dragging with a ceramic tool (drag finish), and vibration treatment (vibratory surface finish) are presented in this paper. The best results were obtained for drag finish. The roughness parameter *Ra* was used to evaluate the roughness. It should be emphasized here that, in conventional machining of machine elements, the roughness parameters *Ra* and *Rz* are used most often to assess surface roughness, because this is related to the path of the cutting edge of the tool on the surface of the workpiece. Usually, these are regular scratches, for example, after turning. The surface of the material created as a result of additive technology is more complex and more parameters characterizing its shape are needed to evaluate it. Surface roughness is just one of the elements of the assessment of the surface layer of elements produced by additive technologies. The quality of the laser-sintered powder is much more complex. Test methods and results for 316L steels are presented in paper [48]. Among other things, surface roughness was investigated, as well as two parameters *Ra* and *Rq*, for samples from 316L steels. Moreover, its corrosion resistance in biomedical applications is presented in [49]. The structure and tendency for corrosion of selectively laser-formed, additive fused (AM) 316 L stainless steel (AM 316L SS), and its wrought counterpart were analyzed. Increased corrosion resistance and improved biological response to 316 L stainless steel were obtained through additive technology.

Taking into account the above considerations, the authors of this article decided to present a wide range of surface roughness assessments, mainly geometric surface structure using modern measurement methods. The tests took into account the printing direction of the elements. The tests were carried out on samples made of 316L steel, which is a well-known and used material, so that the results could be applicable to other users. 

The studies presented herein were performed, in part, within the scope of a grant of the Ministry of Science and Higher Education in Poland concerning construction of an innovative lathe chuck that allows for the compensation of centrifugal force acting on lathe chuck jaws, and thus machining of elements at increased rotational speed of the lathe spindles.

## 2. Materials and Methods 

**Figure 1:**
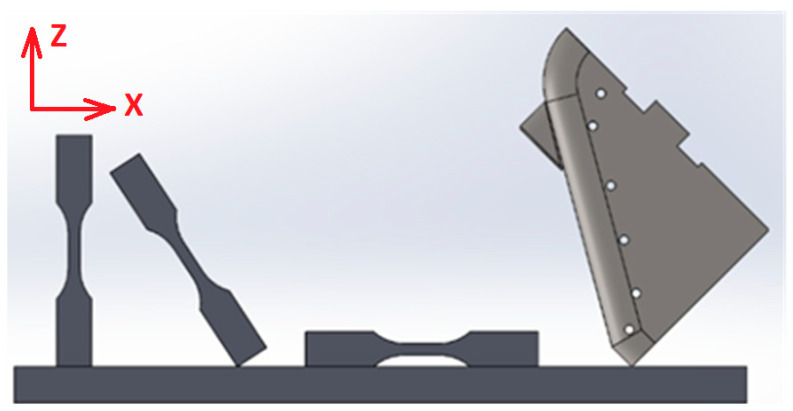
Orientation of samples on the building platform.

**Figure 2:**
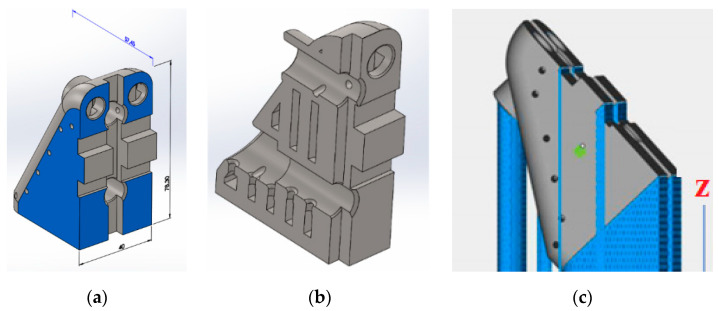
Jaws sample: (**a**) model dimension, (**b**) cross section, and (**c**) samples with support material.

In order to build the sample models, a material based on 316L steel was used. The samples were designed in two variants. The first set of samples was produced in 30 pieces (10 per each direction of printing) according to the standard (ASTM E8/E8M-13 [50]) in order to perform further rheological studies. The second samples were produced in two pieces, and the design is a functional element of the innovative lathe chuck (self-centering chuck jaw). The jaws were manufactured using additive technology in order to produce the unique structure in which the jaws have empty internal cavities that reduce weight, as presented in Figure 1 and Figure 2. 316L steel was also used to build the jaw prototype to avoid corrosion caused by operating fluids in future testing. This type of design (hollow chuck jaws) is unique and does not exist in any other produce lathe chuck. Because of additive technology, it was possible to reduce the lathe jaw weight, a fact that contributes to the reduction of the unfavourable impact of centrifugal force imposed by the jaws’ weight. Sample model shapes were designed using CAD software - SolidWorks (Dassault Systèmes SolidWorks Corp., Waltham, Massachusetts) and saved as STL files (overall dimensions of the sample—40 mm × 78.3 mm × 57.45 mm^3^). Sample models were arranged on the machine working platform in three characteristic orientation variants, as presented in Figure 1.

The samples were produced using the 3D printing machine, CONCEPT LASER M2 (www.concept-laser.de [51], Concept Laser GmbH, Lichtenfels, Germany). The machine has a working space of 250 × 250 × 350 mm^3^ (x, y, z) and is equipped with a laser system having a power of 2 × 200 W. The machine is equipped to use a variety of materials, including 316L, CL 30/31AL, titanium, bronze, and nickel based powders that are used, especially for odontology applications. Upon completion of the manufacturing process, the samples were subjected to an annealing.

**Figure 3:**
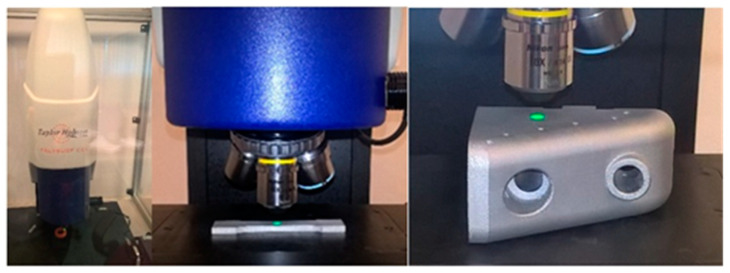
Talysurf CCI Lite—optical profilometer, samples during measurements.

The samples were heated in a furnace to 550 °C for 3 h and then soaked for 6 h to cool outside the furnace. The thickness of built layer (layer height) was 25 µm. Figure 3 presents sample models during metrology measurements using the optical profilometer Talysurf CCI Lite. The examination and determination of the surface texture parameters was performed according to applicable standards (ISO 4287:1997 [52], ISO 25178-2:2012 [53]).

Chemical composition and mechanical properties of steel 316L are presented in Table 1 and Table 2.

During measurements, two-dimensional parameters of surface roughness and spatial parameters were determined. Parameters of measurements were as follows: dimensions of measurement section 1.6 mm × 1.6 mm, number of measurement spots 1024, and applied magnifying lens 10×. In the case of 2D surface roughness parameters, attention was paid to amplitude parameters (*Rp*, *Rv*, *Rz*, *Rc*, *Rt, Ra, Rq, Rsk, Rku*), material ratio (*Rmr*, *Rdc*), and arrangement (*RSm*, *Rdq*). On the basis of the performed measurements, three-dimensional parameters were also determined, among other things, height (*Sq, Ssk, Sku, Sp, Sv, Sz*, and *Sa*), functional (*Smr, Smc*, and *Sxp*), and spatial (*Sal, Str*, and *Std*, as well as *Sdq* and *Sdr*) (ISO 25178-2:2012) [54].

Definitions of some roughness parameters are given below.

- *Sq*—mean square deviation of the surface from the reference surface (root mean square value of the ordinate values within a definition area (*A*)) calculated from the following equation:
  (1) $$Sq=\sqrt{\frac{1}{A}\iint_{A}z^{2}(x,y)dxdy}$$
- *Ssk*—surface asymmetry factor (slant) (quotient of the mean cube value of the ordinate values and the cube of *Sq* within a definition area (*A*)) calculated from the following equation:
  (2) $$Ssk=\frac{1}{Sq^{3}}\left[\frac{1}{A}\sqrt{\iint_{A}z^{3}(x,y)dxdy}\right]$$
- *Sku*—surface slope factor (kurtosis) (quotient of the mean quartic value of the ordinate values and the fourth power of *Sq* within a definition area (*A*)) calculated from the following equation:
  (3) $$Sku=\frac{1}{Sq^{4}}\left[\frac{1}{A}\sqrt{\iint_{A}z^{4}(x,y)dxdy}\right]$$
- *Sp*—largest peak height value within a definition area, *Sv*—minus the smallest pit height value within a definition area, and *Sz*—sum of the maximum peak height value and the maximum pit height value within a definition area calculated from the following equations:
  (4) $$\begin{array}{l} Sp=\sup[z(i,j)]\\ Sv=|\inf[z(i,j)]|\\ Sz=Sp+Sv \end{array}$$
- *Sa*—arithmetic mean of the absolute of the ordinate values within a definition area (A) calculated from the following equation:
  (5) $$Sa=\frac{1}{A}\iint_{A}\left|z(x,y)\right|dxdy$$

Many parameters were used to measure the geometrical surface texture, so that their significance in assessing the surface layer geometry of elements produced by the additive technology could be assessed. In machining, several parameters are usually used to assess the surface, for example, *Ra* and Rz. However, for elements manufactured with additive manufacturing technologies, a simple assessment using two or three parameters may not be sufficient.

## 3. Results

On the basis of the analysis of references and results of previous studies concerning the consumption and technological quality of top layer that depend on technological parameters such as location on the building platform, it was decided to perform complex 2D and 3D measurements together with consideration of statistical analysis. The parameters of surface texture are presented in Table 3 and Table 4. The following elements were determined for each type of positioning on the building platform together with their symbols: *SD*—standard deviation of the test, *U_A_ᵦ*—uncertainty of measurement, and Xᵦ—average value of the test (β—angle of sample inclination against the building platform: 0°, 45°, and 90°). Moreover, for all 30 pieces, total statistical parameters identified with the below symbols were calculated: *X*, *SD*, and *U_A_*. Standard deviation was calculated from Formula (6) and the standard uncertainty using method A from Formula (7). For the sample size *n* = 10, considering Student’s t distribution, the uncertainty was evaluated based on Equation (8).

(6) $$SD=\sqrt{\frac{1}{\left(n-1\right)}\sum_{i=1}^{n}{\left(x_{i}-\bar{x}\right)}^{2}}$$

where *n*—sample size and $\bar{x}$—arithmetical mean of all measured values in a sample.

(7) $$U_{A}=\sqrt{\frac{1}{n(n-1)}\sum_{i=1}^{n}{\left(x_{i}-\bar{x}\right)}^{2}}$$

(8) $$U_{A\beta }=k_{\alpha }\cdot U_{A}$$

where coefficient *k*_α_ is 2.

With the expected value and the standard deviation, it is possible to present the results in the form of normal distribution based on the following formula:

(9) $$f(x)=\frac{1}{SD_{}\sqrt{2\pi }}e^{\frac{-{\left(x_{}-\bar{X}\right)}^{2}}{2SD^{2}}}$$

where *f*(*x*)—probability density function.

**Figure 4:**
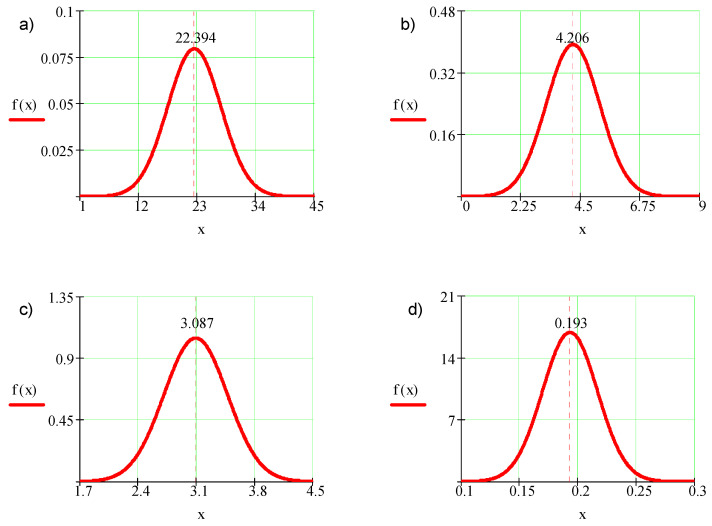
Normal distribution of probability density in the function of the obtained results: (**a**) maximum height of roughness profile—*Rz*, (**b**) mean arithmetic deviation of roughness profile—*Ra*, (**c**) relative material ratio of roughness profile—*Rmr*, and (**d**) mean element width of roughness profile—*Rsm*.

Figure 4 presents exemplary normal distributions of probability density in the function of the obtained results.

On the basis of the calculated statistical parameters and results presented in Graph 4, one may notice a scattering of roughness parameters values.

In order to assess the printing orientation with reference to individual values of surface texture parameters, relative values were introduced related to individual parameters and expressed in percentage. The mean value of a given parameter obtained based on sample surface measurements for all orientations of printing was adopted as the basis; in the considered case, this is the mean from the thirty samples. The mean value of a parameter from measurements concerning individual orientation of printing was referenced to the said basis. This is exemplified by the following Equations (10)–(12):

(10) $$\Delta {\bar{X}}_{0}=\frac{\left|\bar{X}-{\bar{X}}_{0}\right|}{\bar{X}}100\%$$

(11) $$\Delta {\bar{X}}_{45}=\frac{\left|\bar{X}-{\bar{X}}_{45}\right|}{\bar{X}}100\%$$

(12) $$\Delta {\bar{X}}_{90}=\frac{\left|\bar{X}-{\bar{X}}_{90}\right|}{\bar{X}}100\%$$

where $\Delta {\bar{X}}_{0}$, $\Delta {\bar{X}}_{45}$, and $\Delta {\bar{X}}_{90}$—relative value of surface parameters for individual orientations of printing; $\bar{X}$—mean value of a parameter for all orientations of printing$;\;{\bar{X}}_{0}$—mean value of the measured parameters for printing orientation 0°; ${\bar{X}}_{45}$—mean value of the measured parameters for printing orientation 45°; and ${\bar{X}}_{90}$—mean value of the measured parameters for printing orientation 90°. For example, maximum height of the roughness profile *Rz* for printing orientation 0° calculated using Formula (10) based on data from Table 3 would be as follows:

(13) $$\Delta \bar{R}z_{0}=\frac{\left|\bar{R}z-\bar{R}z_{0}\right|}{\bar{R}z}100\%$$

where $\Delta \bar{R}z_{0}$—relative maximum roughness profile height for printing orientation 0°, $\bar{R}z$—mean maximum roughness profile height for all printing orientations (22.4 based on Table 3), and $\bar{R}z_{0}$—mean maximum roughness profile height for printing orientation 0° (21.2 based on Table 3).

**Figure 5:**
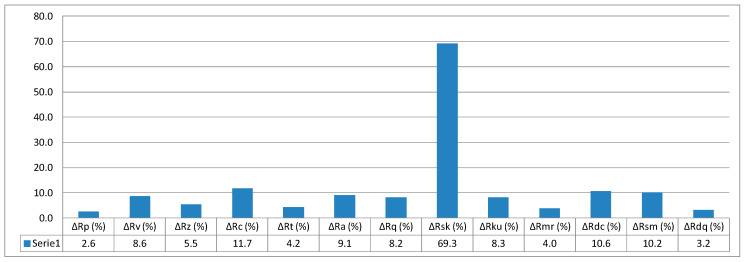
Relative values of surface parameters for printing orientation 0°.

**Figure 6:**
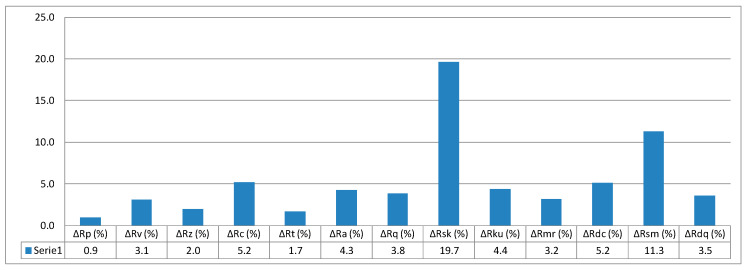
Relative values of surface parameters for printing orientation 45°.

**Figure 7:**
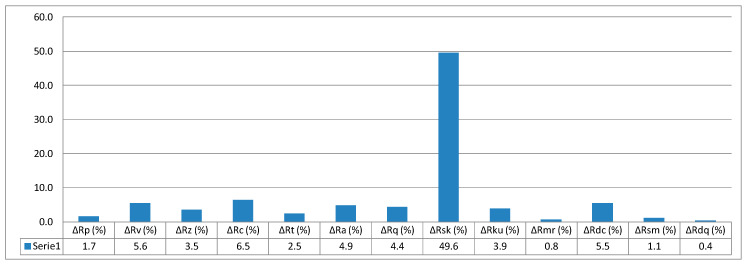
Relative values of surface parameters for printing orientation 90°.

The value calculated based on Formula (13) is 5.5%. In this way, it is possible to determine all relative values of surface parameters with reference to printing orientation. The relative values of individual parameters are given in column graphs, Figure 5, Figure 6 and Figure 7.

When quantitatively analysing the results of the study, it can be said that there are distinct differences in the obtained surface texture parameter values depending on the models’ orientation on the building platform. Concerning two-dimensional roughness parameters, the most advantageous orientation variant showing the least parameter values such as mean arithmetic deviation of roughness profile from mean line *Ra*; maximum profile height *Rz*; total profile height *Rt, Rv*; and other *Rq, Rdc*, and so on is the orientation at the angle of 0°, in other words, parallel to the base plane of the machine’s working space. Ten out of thirteen measured parameters for the angle 0° show values less than mean values (measured for 30 samples) from 2.6% to 11.7%. Roughness profile asymmetry *Rsk* parameter shows the most significant differences from the printing orientation standpoint. This results from the fact that this parameter is inversely proportional to the cube of other roughness parameter *Rq* and is the third-order moment of the amplitude distribution curve determined along the measurement section. As the references show, these parameters directly affect the consumption of mating machine elements.

**Figure 8:**
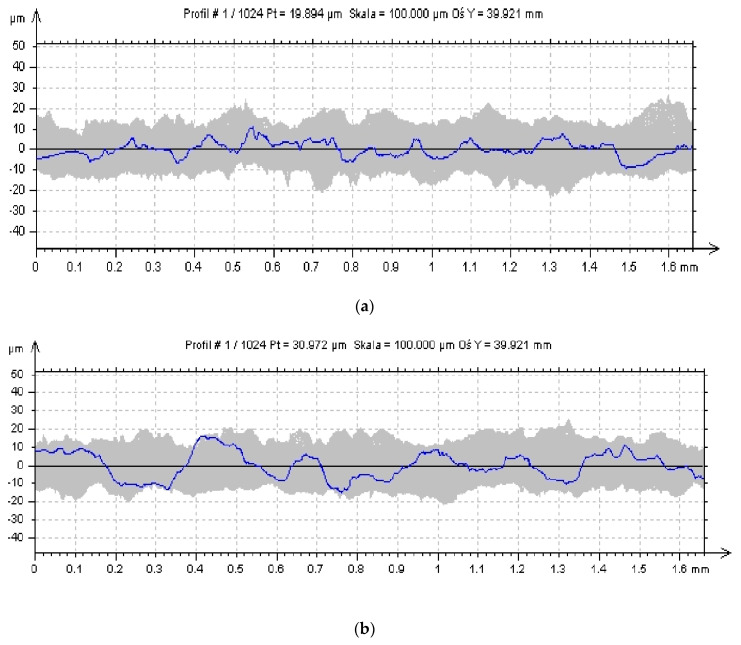

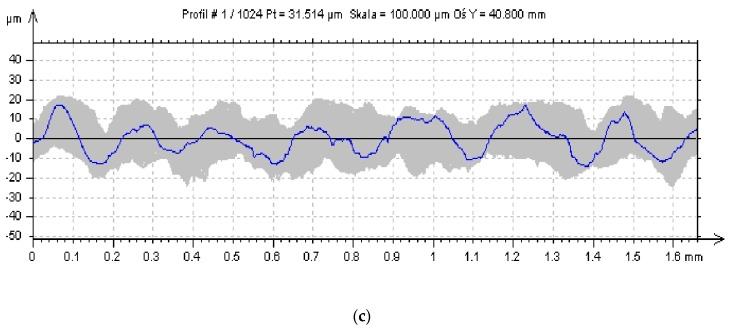
2D roughness profile for samples: (**a**) 0°, (**b**) 45°, and (**c**) 90°.

Figure 8 presents surface roughness profiles for three sample models’ orientation variants.

**Figure 9:**
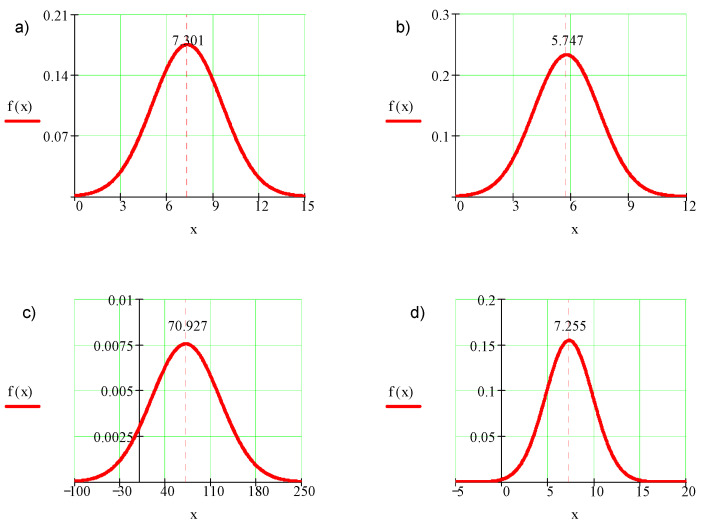
Normal distribution of probability density in the function of the obtained results: (**a**) height of Rms surface—*Sq*, (**b**) mean arithmetic surface height—*Sa*, (**c**) orientation of surface height—*Std*, and (**d**) developed ratio of surface interphasic area—*Sd*.

Figure 9 presents the exemplary normal distribution of probability density in the function of the obtained results given in Table 4, calculated based on Formula (4).

**Figure 10:**
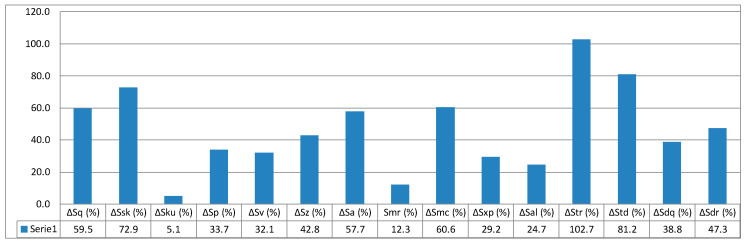
Relative values of surface parameters for printing orientation 0°.

**Figure 11:**
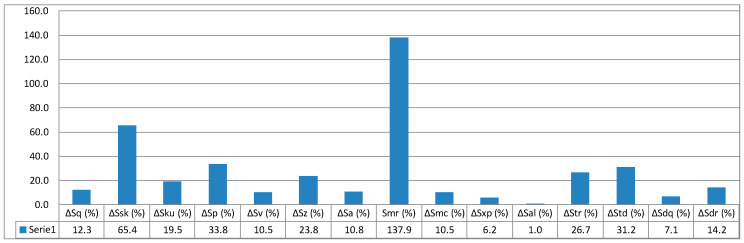
Relative values of surface parameters for printing orientation 45°.

**Figure 12:**
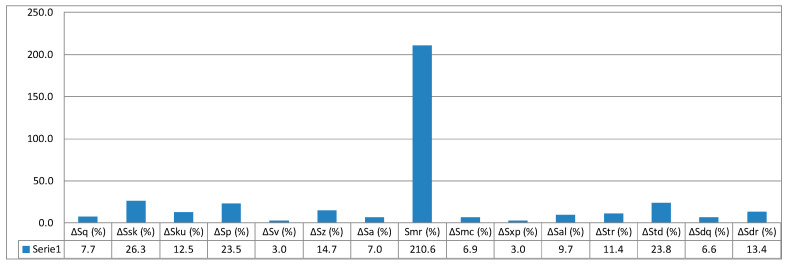
Relative values of surface parameters for printing orientation 90°.

Relative values of individual surface geometrical parameters calculated based on formulae (5, 6, 7) and data given in Table 4 are presented in a column graph in Figure 10, Figure 11 and Figure 12.

Quantitative analysis of 3D spatial parameters measurement results, concerning height-related, functional, spatial, and hybrid, affirm what is similar to the case of 2D flat parameters of roughness: there are distinct differences in measurement results. In the case of samples measurements performed at a given angle 45° and 90°, mean values of parameters are in almost all cases less than mean values for the 30 samples. The only exception is the surface texture orientation parameter, wherein the values are less than mean values. For samples produced at the set angle 0°, orientation expressed with the *Std* parameter has values less than average. The value of the parameter is expressed in degrees, which means a different nature of surface texture, 0–13.3°, 45–93.1°, and 90–87.8°, respectively.

**Figure 13:**
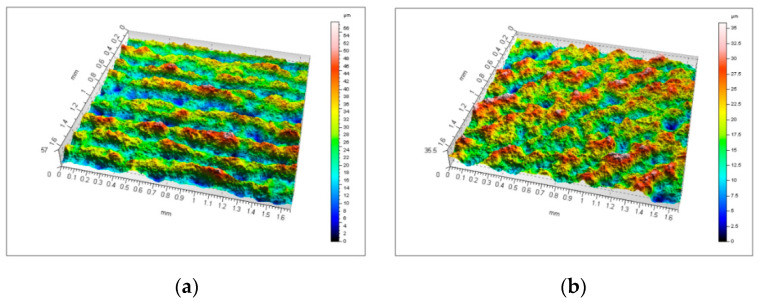

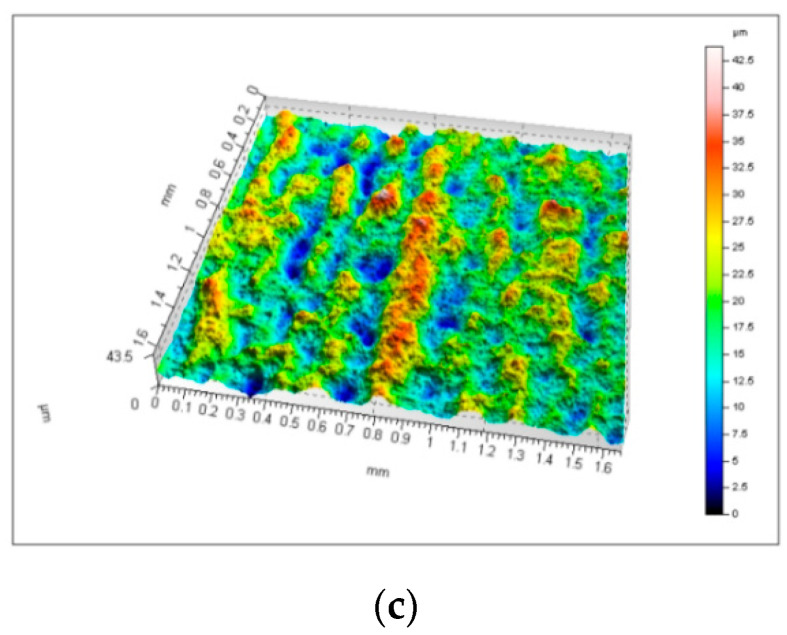
3D roughness profile for samples: (**a**) 0°, (**b**) 45°, and (**c**) 90°.

Figure 13 presents isometric views of geometrical texture of the examined surface. Attention must be paid to the functional parameter of surface material ratio — *Smr*. It is responsible for the surface roughness share expressed in percentage and shows the greatest relative deviations for the angles 45° and 90° at 137.9% and 210.6%, respectively, which can be particularly useful for assessing the impact of print orientation (print direction) on surface roughness quality. All values of spatial parameters for orientation at an angle of 0° show significant differences from the values of parameters measured for directions 45° and 90°.

Owing to the layered nature of the additive manufacturing process, the images (Figure 13) show both deep valleys and high peaks, which is typical for the so-called step effect. This roughness is regular and corresponds to the angles of sample orientation on the working platform.

The test results presented above are an attempt to identify the geometrical texture of the surface of elements manufactured by additive technology—powder bed fusion. The surface texture of elements produced by methods known to date, for example, turning or milling, is quite well known and described in the literature. In mechanical engineering, several basic roughness parameters are usually used to describe it (*Ra, Rz, Rt*), usually only 2D parameters. In the case of a surface obtained by additive technology—as in the presented research results—one or two 2D parameters are not enough; it is necessary to use more complex tools and research methods (optical) enabling measurement of the geometrical surface texture—3D and identification of 3D parameters, giving much more information about the surface compared with the parameters of a single profile. In technological cases in which it is not possible to perform finishing treatment, for example, grinding, and the obtained surface can affect the functionality of the elements, a thorough knowledge of its geometric texture is of key importance. This may apply to different micro-mechanisms or, for example, micro-channels. Only the correct identification of the surface geometry structure, which has been presented above, enables further research, for example, in terms of its impact on various processes.

## 4. Conclusions

When analysing the above-mentioned study results and the state-of-the-art references, the following general conclusions can be derived.

The arrangement of sample models on the working platform of the machine affects the value of all tested parameters of surface texture.

The most advantageous variant of model orientation is the case where the surface is parallel to the building platform plane (samples orientation variant—1). The values of height parameters of surface roughness (2D) for individual orientations differ from the average value obtained for all orientations in the range from 0.4% to 69.3%. On the basis of normal distribution of probability density and values of included roughness (*Ra, Rz*), it can be said that they are close to the values of these parameters obtained during conventional machining (milling, turning).

On the basis of the spatial parameters value of the surface texture, providing a full image of the surface texture quality (3D image), it is possible to claim that the measurement results scattering presented in normal distributions are clearly greater for the *Std* parameter, which characterizes the orientation of the surface texture. At the same time, the graph shows the least value of the probability density function. The values of spatial surface roughness (3D) parameters for individual orientations differ from the average value obtained for all orientations in the range from 1% to 210.6%, while for the 0° orientation, these differences are greater than for other cases.

In summary, it is necessary to emphasize that, in the case of contact additive technologies, application of surface texture parameters measurements and their narrowing to the analysis of typical amplitude parameters, commonly applied in the industry, is not sufficient. The nature of the art allows for performing complete three-dimensional surface texture analysis, as suggested by the authors in the case of the studies of models manufactured using additive technologies. Understanding the impact of the orientation of the workpiece on the 3D printer’s working platform on surface roughness parameters is important when planning allowances for further processing, for example, by grinding. Therefore, machining allowances should be planned depending on the orientation on the working platform, and this may especially apply to the processing of various micro parts. Identification of the geometric texture of the surface presented in this work can be particularly useful in cases where it is not possible to perform finishing technological operations, for example, on internal surfaces of elements (hollow). In these cases, 0° orientation can be recommended. Differences in the geometric texture of the surface may affect the fatigue strength of the elements, as well as their tribological wear, which may depend on the orientation of the models on the machine’s working platform. The determination of specific values requires additional research, which the authors plan to do in the future.

Taking into account the results of surface texture research and the literature analysis, it can be concluded that 3D printing has very high potential applications and, in the near future, owing to the continuous development of technology, their precision, and resolution affecting the quality of the surface layer, it will be possible to produce full finished final models for MEMS technology.

## Figures and Tables

**Table 1:** Chemical composition of steel 316L.

Component	Cr	Ni	Mo	Mn	Si	P	C	S	Fe
Indicative value, %	16.5–18.5	10.0–13.0	2–2.5	0–2.0	0–1.0	0–0.045	0–0.030	0–0.030	Balance

**Table 2:** Mechanical properties of steel 316L.

Properties	90°, Upright	45°, Polar Angle	0°, Horizontal
Yield strength, R_p0,2_	374 ± 5 N/mm^2^	385 ± 6 N/mm^2^	330 ± 8 N/mm^2^
Tensile strength, Rm	650 ± 5 N/mm^2^	640 ± 7 N/mm^2^	529 ± 8 N/mm^2^
Elongation, A	(65 ± 4)%	(63 ± 5)%	(63 ± 5)%
Young’s modulus	ca. 200 × 10^3^ N/mm^2^		
Hardness	20 HRC		

**Table 3:** Value of the 2D surface roughness parameters.

Sample Number	*Rp*, (µm)	*Rv*, (µm)	*Rz*, (µm)	*Rc*, (µm)	*Rt*, (µm)	*Ra*, (µm)	*Rq*, (µm)	*Rsk*	*Rku*	*Rmr*, (%)	*Rdc*, (µm)	*RSm*, (µm)	*Rdq*, (^0^)
1	24.6	19.8	44.4	27.3	52.3	8.4	10.4	0.491	3.479	0.9	17.0	0.228	25.0
2	11.9	9.5	21.5	11.7	25.6	3.8	4.8	0.41	3.833	1.0	7.6	0.178	15.0
3	12.2	9.0	21.2	11.0	26.9	3.6	4.6	0.648	4.54	0.9	7.2	0.171	14.9
4	8.5	8.3	16.8	9.0	19.2	3.0	3.7	−0.007	2.943	1.1	6.4	0.16	13.1
5	9.4	8.5	17.9	9.4	20.5	3.1	3.9	0.167	3.129	1.1	6.6	0.15	14.4
6	7.9	8.1	16.0	9.0	18.2	2.9	3.6	−0.027	2.821	1.4	6.3	0.164	12.7
7	9.3	9.1	18.4	10.1	21.0	3.4	4.1	0.041	2.964	1.3	7.1	0.167	13.8
8	9.5	8.1	17.6	9.6	20.5	3.1	3.9	0.34	3.337	1.3	6.5	0.171	12.9
9	10.4	8.8	19.2	10.5	22.6	3.5	4.3	0.229	3.18	1.2	7.3	0.174	14.0
10	9.4	9.0	18.5	10.2	21.5	3.3	4.1	0.087	3.197	1.2	7.1	0.171	13.9
${\bar{x}}_{0}$	11.3	9.8	21.2	11.8	24.8	3.8	4.8	0.238	3.342	1.1	7.9	0.173	15.0
SD	4.9	3.5	8.3	5.5	10.0	1.6	2.0	0.228	0.512	0.2	3.2	0.021	3.6
**U_A0_**	1.5	1.1	2.6	1.7	3.2	0.5	0.6	0.072	0.162	0.1	1.0	0.007	1.1
11	9.2	9.6	18.8	11.4	21.7	3.6	4.4	−0.071	2.923	1.5	7.6	0.199	12.4
12	13.7	12.0	25.7	16.2	29.4	4.9	6.0	0.22	2.962	1.1	10.3	0.222	14.9
13	13.4	11.1	24.5	15.3	29.9	4.9	6.0	0.445	3.481	1.3	10.2	0.234	14.1
14	12.1	11.2	23.4	14.7	26.7	4.7	5.7	0.149	2.771	1.2	10.1	0.23	13.9
15	11.4	10.8	22.3	13.3	24.7	4.2	5.1	0.059	2.724	1.2	8.8	0.208	14.0
16	12.1	12.6	24.7	15.7	28.7	4.8	5.9	−0.028	2.735	1.1	10.4	0.232	14.3
17	11.8	11.4	23.1	15.0	26.2	4.5	5.5	0.081	2.727	1.2	9.6	0.223	14.0
18	10.4	10.1	20.5	11.8	23.4	3.8	4.7	0.048	3.08	1.2	7.8	0.183	14.3
19	11.3	10.5	21.8	12.4	25.8	4.1	5.0	0.099	3.238	1.2	8.5	0.205	13.4
20	12.0	11.6	23.5	14.4	27.1	4.6	5.6	0.127	2.871	1.2	9.7	0.212	14.6
${\bar{x}}_{45}$	11.7	11.1	22.8	14.0	26.4	4.4	5.4	0.113	2.951	1.2	9.3	0.215	14.0
SD	1.3	0.9	2.1	1.7	2.6	0.5	0.6	0.143	0.251	0.1	1.0	0.016	0.7
**U_A45_**	0.4	0.3	0.7	0.5	0.8	0.1	0.2	0.045	0.079	0.0	0.3	0.005	0.2
21	11.5	10.1	21.6	12.9	25.3	4.1	5.0	0.282	3.172	1.2	8.5	0.176	14.5
22	13.7	13.7	27.4	16.7	31.5	5.1	6.3	−0.01	2.97	1.0	10.6	0.203	15.8
23	11.9	12.5	24.5	15.4	27.3	4.7	5.7	−0.066	2.733	1.3	10.2	0.194	15.3
24	11.4	10.0	21.4	12.8	24.2	4.1	5.0	0.153	2.756	1.1	8.8	0.184	14.9
25	11.7	10.8	22.5	14.0	26.3	4.4	5.3	0.099	3.199	1.2	9.1	0.204	14.4
26	11.7	12.4	24.1	15.7	27.3	4.7	5.7	−0.105	2.747	1.2	10.2	0.195	14.6
27	12.1	10.5	22.6	12.4	25.5	4.0	5.0	0.247	3.213	1.0	8.2	0.178	14.5
28	11.0	10.1	21.2	12.6	23.8	3.9	4.8	0.162	2.966	1.3	8.1	0.182	13.1
29	11.0	10.5	21.5	12.6	24.8	4.0	5.0	0.046	2.903	1.2	8.5	0.183	13.4
30	12.1	13.1	25.2	16.8	29.8	5.2	6.2	−0.1	3.016	1.4	11.2	0.209	15.1
${\bar{x}}_{90}$	11.8	11.4	23.2	14.2	26.6	4.4	5.4	0.071	2.968	1.2	9.3	0.191	14.6
SD	0.8	1.4	2.0	1.8	2.5	0.5	0.5	0.140	0.186	0.1	1.1	0.012	0.8
**U_A90_**	0.2	0.4	0.6	0.6	0.8	0.1	0.2	0.044	0.059	0.0	0.4	0.004	0.3
$\bar{x}$	11.6	10.8	22.4	13.3	25.9	4.2	5.2	0.141	3.087	1.2	8.9	0.193	14.5
SD	2.8	2.3	5.0	3.5	6.0	1.0	1.3	0.1841	0.3814	0.1	2.1	0.0236	2.1
**U_A_**	0.5	0.4	0.9	0.6	1.1	0.2	0.2	0.0336	0.0696	0.0	0.4	0.0043	0.4

**Table 4:** Value of the 3D surface roughness parameters.

Sample Number	*Sq*, (µm)	*Ssk*	*Sku*	*Sp*, (µm)	*Sv*, (µm)	*Sz*, (µm)	*Sa*, (µm)	*Smr*, (%)	*Smc*, (µm)	*Sxp*, (µm)	*Sal*	*Str*	*Std*,°	*Sdq*	*Sdr*, (%)
1	17.1	1.085	4.381	70.8	42.1	113.0	13.2	0.01	22.5	20.5	0.109	0.389	26.499	0.68	19.9
2	8.7	1.182	8.776	66.1	24.2	90.2	6.5	0.012	10.3	14.5	0.083	0.1	3.974	0.433	8.6
3	7.4	1.272	6.821	42.1	20.0	62.0	5.4	0.013	8.6	11.3	0.065	0.444	153.521	0.43	8.5
4	5.7	0.005	3.008	24.6	21.1	45.6	4.6	0.004	7.4	11.3	0.054	0.107	0.146	0.374	6.6
5	5.9	0.039	2.95	34.9	23.1	58.0	4.8	0.001	7.7	11.5	0.049	0.059	0.099	0.417	8.1
6	5.7	0.146	2.989	23.1	21.8	44.9	4.5	0.003	7.4	10.8	0.052	0.13	0.12	0.37	6.5
7	8.4	0.229	2.703	32.3	25.4	57.7	6.9	0.002	11.1	14.8	0.05	0.061	0.076	0.436	8.9
8	8.4	0.703	3.74	36.0	22.9	58.8	6.6	0.013	11.1	12.7	0.08	0.096	4.43	0.388	7.0
9	12.2	1.114	4.258	47.4	28.7	76.1	9.2	0.016	17.5	15.1	0.118	0.142	8.701	0.413	8.0
10	6.2	0.214	3.108	27.2	22.5	49.7	4.9	0.007	8.0	11.5	0.054	0.355	0.143	0.403	7.6
${\bar{x}}_{0}$	11.6	0.650	3.745	49.0	32.3	81.3	9.1	0.009	15.3	16.0	0.082	0.372	13.321	0.542	13.8
SD	3.6	0.524	1.997	16.6	6.4	21.7	2.7	0.005	5.0	3.0	0.025	0.147	47.695	0.089	3.9
**U_A0_**	2.3	0.332	1.263	10.5	4.1	13.7	1.7	0.003	3.2	1.9	0.016	0.093	30.165	0.057	2.5
11	5.4	−0.018	2.939	17.0	18.9	35.9	4.3	0.035	7.1	10.7	0.062	0.209	51.199	0.335	5.3
12	7.3	0.353	3.169	39.8	24.4	64.2	5.8	0.008	9.8	12.5	0.06	0.051	135.034	0.393	7.3
13	8.2	1.034	5.137	43.7	20.3	64.0	6.3	0.002	10.1	11.6	0.07	0.127	26.462	0.386	7.1
14	7.0	0.26	2.734	25.4	40.6	66.0	5.7	0.005	9.5	12.1	0.058	0.093	134.985	0.377	6.7
15	6.4	0.14	2.924	26.1	21.5	47.6	5.3	0.002	8.4	12.1	0.063	0.177	134.998	0.374	6.6
16	7.4	0.192	3.134	32.0	23.9	55.9	5.9	0.004	9.5	14.0	0.058	0.053	116.483	0.375	6.6
17	6.8	0.229	2.759	26.4	39.7	66.1	5.5	0.002	9.3	11.9	0.062	0.085	135.007	0.377	6.7
18	5.9	−0.039	3.209	22.3	26.9	49.2	4.7	0.005	7.5	11.7	0.052	0.256	47.899	0.385	7.0
19	6.5	0.378	3.407	29.2	20.1	49.2	5.1	0.002	8.3	11.7	0.065	0.255	141.207	0.363	6.2
20	7.4	0.278	2.799	26.0	24.9	50.9	5.9	0.001	9.9	12.6	0.07	0.06	134.966	0.39	7.1
${\bar{x}}_{45}$	6.4	0.130	2.869	21.5	21.9	43.4	5.1	0.018	8.5	11.6	0.066	0.135	93.083	0.363	6.2
SD	1.4	0.209	0.099	6.4	4.3	10.6	1.1	0.024	2.0	1.3	0.006	0.105	59.232	0.039	1.3
**U_A45_**	0.9	0.132	0.063	4.0	2.7	6.7	0.7	0.015	1.3	0.8	0.004	0.067	37.462	0.025	0.8
21	5.9	0.422	3.385	24.1	22.5	46.6	4.7	0.022	7.9	10.1	0.058	0.135	85.581	0.363	6.2
22	7.3	0.303	3.106	28.4	23.2	51.6	5.8	0.001	9.4	12.8	0.058	0.07	93.487	0.381	6.8
23	6.8	0.078	2.99	24.5	25.4	49.9	5.4	0.009	8.9	12.6	0.057	0.398	93.759	0.377	6.7
24	5.9	0.226	2.828	24.0	18.8	42.8	4.8	0.001	7.8	10.5	0.065	0.148	89.969	0.377	6.7
25	6.6	0.487	4.254	54.8	20.6	75.4	5.2	0	8.3	11.8	0.075	0.411	89.996	0.368	6.3
26	6.8	0.074	3.081	25.1	28.5	53.6	5.4	0.008	8.9	12.8	0.058	0.33	63.504	0.358	6.1
27	6.2	0.336	3.296	24.1	19.6	43.6	4.9	0.007	8.1	11.2	0.078	0.181	90.037	0.362	6.2
28	5.8	0.315	3.205	26.7	18.8	45.5	4.6	0.001	7.6	10.2	0.058	0.238	85.563	0.324	5.0
29	5.8	0.113	2.988	25.2	18.6	43.9	4.6	0.006	7.6	11.1	0.06	0.072	89.973	0.327	5.1
30	7.5	0.132	2.856	25.6	24.9	50.6	6.0	0.025	9.8	13.9	0.06	0.274	89.999	0.366	6.4
${\bar{x}}_{90}$	6.7	0.277	3.121	24.9	23.7	48.6	5.3	0.024	8.8	12.0	0.059	0.205	87.790	0.365	6.3
SD	1.1	0.205	0.374	1.1	1.7	2.8	1.0	0.002	1.3	2.7	0.001	0.098	3.124	0.002	0.1
**U_A90_**	0.7	0.130	0.237	0.7	1.1	1.8	0.6	0.001	0.8	1.7	0.001	0.062	1.976	0.001	0.1
$\bar{x}$	7.3	0.376	3.564	32.5	24.5	57.0	5.7	0.008	9.5	12.4	0.065	0.184	70.927	0.390	7.3
SD	2.3	0.381	1.306	12.9	6.2	15.7	1.7	0.008	3.1	2.0	0.016	0.123	52.658	0.061	2.6
**U_A_**	0.4	0.070	0.238	2.3	1.1	2.9	0.3	0.001	0.6	0.4	0.003	0.022	9.614	0.011	0.5
